# Justification for hysterectomies and frequency of histopathological lesions of hysterectomy at a Teaching Hospital in Peshawar, Pakistan

**DOI:** 10.12669/pjms.291.2509

**Published:** 2013

**Authors:** Arzoo Amin, Azmat Ali, Zohra Amin, Farah Nighat Sani

**Affiliations:** 1 Dr. Arzoo Amin, MCPS, FCPS, Assistant Professor, Dept. of Obstetrics and Gynecology, Peshawar Medical College, Peshawar, Pakistan.; 2 Dr. Azmat Ali, MS, Neurosurgeon, Pakistan Institute of Medical Sciences Islamabad.; 3 Ms. Zohra Amin, Final Year Student (MBBS), Mardan Medical Complex, Peshawar. Pakistan.; 4 Dr. Farah Nighat Sani, MCPS, Department of Obstetrics and Gynecology, Mardan Medical Complex, Peshawar. Pakistan.

**Keywords:** Complications, Histopathology, Hysterectomy

## Abstract

***Objective: ***To determine the justification for hysterectomies and the frequencies of histopathological lesions and complications in hystrectomised patients.

***Methodology: ***As a part of a quality assurance process at the Mercy Teaching Hospital, hysterectomies performed between 1^st^ January, 2010 and 1^st^ Jan 2012 were retrospectively analyzed for presenting complaints, surgical indication, histologic findings, and postoperative complications. The hysterectomy was considered justified if the preoperative diagnosis was verified by the pathology report or if significant alternate pathology was present.

***Results: ***A total of 123 hysterectomies were performed during this period. Eleven (8.9%) patients’ results could not be traced. The other 91.1% had some pathology found. Histologic findings reconfirmed the clinical diagnoses. The hysterectomies were considered justified if p=0.000. Hysterectomy was performed abdominally in 88 (71.5%) patients, vaginally in 35 patients (28.4%). The most common indication for hysterectomy was fibroid related menorrhagia n=40(32.5%), followed by third degree uterovaginal prolapse n=30(24.4%), and dysfunctional uterine bleeding 29(23.6%) patients. Fever was the most common 7(5.7%) post operative complication followed by urinary tract infection 5(4.9%) The incidence of postoperative fever was greater following abdominal surgery, while urinary tract infection was greater following vaginal hysterectomy (P=0.370).

***Conclusion: ***Almost 91.1% of all hysterectomies in this study were justified. Clinical diagnoses were related to presenting complaints (p=0.000) and were confirmed by histopathogic findings (p=0.000). Most of the hysterectomies were carried out abdominally in part because fewer patients presented with prolapse.

## Introduction

 The most common major surgical procedure performed in Gynecology is Hysterectomy.^1^ Many medical and conservative surgical treatment options are available but still hysterectomy remains the most common gynecological procedure performed world wide.^2^

 In response to the consistent demand for this procedure, hysterectomy has been identified as a key health care indicator in recent reports, to measure and compare hospital performance.^3^ Although hysterectomy is usually done to improve patient’s quality of life yet it has its own morbidity and mortality.^4^ Most of the indications are debatable and therefore regular audit of this should be done. Due to non availability of population based studies providing estimates of hysterectomy prevalence and histopathological analysis of hysterectomies, in Pakistan there has always been concern about its high prevalence.

 This study was designed to analyze relationship between clinical indication for hysterectomy and postoperative histological findings, and to audit complications of hysterectomy at the Gynecologic Surgical Unit of Mercy Teaching Hospital Peshawar.

## Methodology

 This is a retrospective study conducted at the department of obstetrics and gynecology Mercy Teaching Hospital and department of Pathology Peshawar Medical Collage, Peshawar. Records from history sheets and files of patient admitted in the gynecology ward for hysterectomy during the last two years from 1st January 2010 up to 1^st^ January 2012 were collected. Obstetrical hysterectomies were excluded from the study. Information was gathered regarding age, parity, clinical diagnoses, presenting complaint, menstrual history and preoperative diagnosis/indications of hysterectomy and any complications found. Histopathology reports of some patients were collected from the department of pathology and their diagnosis noted. Data was analyzed by using percentages.

## Results

 The majority of patients (41.5%) were in the age range of 46-55 years, although the age range was from 35-65 years, 93 (75.6%) had parity >5. The most common presenting complaints were excessive and irregular menstrual blood loss in 65 (52%) patients, followed by something coming out of the vagina in 37 (30%) patients. Other complaints were abdominal mass in 12 (9.8%) patients and post menopausal bleeding in 4 (3.3%) and mentally retarded only one patient.

 Pre-operative diagnosis of fibroid was made in 40 (32.5%) patients, followed by third degree uterovaginal (uv) prolapse in 30 (24.4%), dysfunctional uterine bleeding in 29 (23.6%) patients and 7 (5.7%) had second degree uv prolapse. Seven patients (5.7%) had malignancy [5 endometrial carcinoma(ca)one cervical and one ovarian ca] suspected which was then confirmed on histopathological findings as well.

 A total of 123 hysterectomies were performed during this period. Hysterectomy was performed abdominally in 88 (71.5%) patients, vaginally in 35 patients (28.4%). The most common indication was fibroid-related menorrhagia in 40 (32.5%) patients, followed by third degree uterovaginal prolapse in 30(24.4%) patients, and dysfunctional uterine bleeding in 29 (23.6%) patients. Although 100 (81.3%) patients had no complications, among the 23 (18.7%) who had post operative complications, fever was the most common in 7(5.7%) patients followed by urinary tract infection in 5(4.9%) patients. The incidence of postoperative fever was greater following abdominal rather than vaginal surgery, while urinary tract infection was greater following vaginal rather than abdominal (P=0.370). Eleven (8.9%) patients’ histopathology results could not be traced. The other 91.1% had some pathology identified.

 Histologic findings reconfirmed the clinical diagnoses, with statistically significant results (p=0.000) and the hysterectomies was considered justified. Clinical diagnoses were significantly related to presenting complaints (p=0.000). Chronic non specific cervicitis was found more common in those having second degree or third degree prolapsed, 17 (13.8%). Clinical diagnoses was significantly related with presenting complaints (p=0.000), age (p=0.000) and histopathology (p=0.000). There was no mortality in this duration among hysterectomy patients.

## Discussion

 The most common presenting complaints were excessive and irregular menstrual blood loss in 65 (52%) patients, followed by something coming out of vagina in 37 (30%) patients. The most common indication for hysterectomy was fibroid uterus followed by uterovaginal prolapsed. Similar results were seen by Qamar-un-Nisa^2^ et al at Muhammad Medical College hospital, Mirpurkhas and Iftekhar R et at^5^ Karachi where menorrhagia was the most common complaint because of fibroid uterus 30% and the most common histopathological finding was fibroid uterus 30% followed by chronic non specific cervicitis. Other studies by Weaver et al^6^, Sarfaraz^7^, Westphalen JB^8^ have also reported liomyoma as the most common pathological lesion with the frequencies of 25%, 31% and 15.32% respectively. However Ahsan et at^9^, at Liaquat National Hospital Karachi, found adenomyosis to be the most common pathology followed by liomyoma. This is in contrast to the findings of the study done by Qamar-un-Nisa^2^, Iftekhar^5^ and Bukhari^10^ where chronic nonspecific cervicitis was the common histopathological diagnoses.

 Most hysterectomies were carried out abdominally rather than vaginally, in part because fewer patients presented with prolapse. This is similar to the findings of the study done by Fayaz S^11^ and Jennifer.^12^

 One of the patients was under the age of 20 years and the hysterectomy was performed because she was mentally retarded and with parent’s insistence she was operated upon. Ninety-three (75.6%) patients had parity >5. Two (1.6%) infertile patients had hysterectomy done and one of them was having endometrial Ca at the age of 40 years while the other one had 16 cm large size fibroid at the age of 48 years with severe menorrhagia.

 The incidence of postoperative fever and urinary retention was greater following abdominal rather than vaginal surgery, while urinary tract infection was greater following vaginal rather than abdominal (P=0.370). This is in contrast to study done by Kafy S^13^ in Canada where more retention occurred in vaginal group. This is similar to study done by Fayaz S^11^ in which more complications were associated with abdominal hysterectomy. Complications were not significantly related to age, parity and diagnoses. This is in contrast to the findings of the study done by Bashir^1^ in which these were related to complications.

## Conclusions

 In general 91.1% of all hysterectomies in this study were justified. Clinical diagnoses were related to presenting complaints (p=0.000) and were confirmed by histopathogic findings (p-0.000). Hysterectomy proved curative and acceptable form of therapy to most of the patients. A yearly audit should be done in every institute to collect data and to analyze the pattern of indications, histopathological lesions and complications of hysterectomy.

## References

[1] Bashir R, Parveen Z, Sultana R, Khan B (2005). A two years audit of complications of hysterectomy at Ayub Teaching Hospital Abbottabad. J Ayub Med Coll Abbottabad.

[2] Qamar-ur-Nisa, Hemlata, Habibullah, Memon F, Shaikh TA, Memon Z (2011). Hysterectomies: An audit at a tertiary care hospital. Prof Med J.

[3] Toma A, Hopman WM, Gorwill RH (2004). Hysterectomy at a Canadian tertiary care facility: results of a one year retrospective review. BMC Women Health.

[4] Olsson JH, Ellstrom M, Hahlin M (1996). A randomized prospective trail comparing laparoscopic and abdominal hysterectomy. Br J Obstet Gynecol.

[5] Iftikhar R (2005). The outcome of subtotal abdominal hysterectomy. J Coll Physicians Surg Pak.

[6] Weaver F, Hynes D, Goldberg JM (2001). Hysterectomy in Veterans Affairs Medical Centres. Obstet Gynecol.

[7] Sarfraz T, Tariq H (2005). Hitopathological findings in menorrhagia. a study of 100 hysterectomy specimens. Pak J Pathol.

[8] Westphalen JB, Fraser S, Rea EE, Coulthard AC, Ng KH, Walters WA (1992). Medical audit of hysterectomy in the Hunter Area of New South Wales. Aust Clin Rev.

[9] Ahsan S, Naeem S, Ahsan A (2001). A case note analysis of hysterectomies performed for non neoplastic indications Liaquat National Hospital, Karachi. J Pak Med Assc.

[10] Bukhari U, Sadiq S (2007). Analysis of the underlying pathological lesions in hysterectomy specimens. Pak J Pathol.

[11] Fayyaz S, Majid SS (2001). Audit of Gynecological hysterectomies. JPMI.

[12] Butt JL, Jeffery ST, Van derSpuyZM (2012). An audit of indications and complications associated with elective hysterectomy at a public service hospital in South Africa. Int J Gynecol Obstet.

[13] Kafy S, Huang J, AlSunaidi M, Weiner D, Tulanadi T (2006). Audit of Morbidity and Mortality Rate of 1792 Hysterectomies. J Minimally Invasive Gynaecol.

